# Mapping the Intramolecular Communications among Different Glutamate Dehydrogenase States Using Molecular Dynamics

**DOI:** 10.3390/biom11060798

**Published:** 2021-05-27

**Authors:** Shaherin Basith, Balachandran Manavalan, Tae Hwan Shin, Gwang Lee

**Affiliations:** 1 Department of Physiology, School of Medicine, Ajou University, Suwon 16499, Korea; shaherinb@gmail.com (S.B.); bala@ajou.ac.kr (B.M.); catholicon@ajou.ac.kr (T.H.S.); 2 Department of Molecular Science and Technology, Ajou University, Suwon 16499, Korea

**Keywords:** glutamate dehydrogenase, enzyme, molecular dynamics, network analysis, allostery, regulation, modulators

## Abstract

Glutamate dehydrogenase (GDH) is a ubiquitous enzyme that catalyzes the reversible oxidative deamination of glutamate to α-ketoglutarate. It acts as an important branch-point enzyme between carbon and nitrogen metabolisms. Due to the multifaceted roles of GDH in cancer, hyperinsulinism/hyperammonemia, and central nervous system development and pathologies, tight control of its activity is necessitated. To date, several GDH structures have been solved in its closed form; however, intrinsic structural information in its open and apo forms are still deficient. Moreover, the allosteric communications and conformational changes taking place in the three different GDH states are not well studied. To mitigate these drawbacks, we applied unbiased molecular dynamic simulations (MD) and network analysis to three different GDH states i.e., apo, active, and inactive forms, for investigating their modulatory mechanisms. In this paper, based on MD and network analysis, crucial residues important for signal transduction, conformational changes, and maps of information flow among the different GDH states were elucidated. Moreover, with the recent findings of allosteric modulators, an allosteric wiring illustration of GDH intramolecular signal transductions would be of paramount importance to obtain the process of this enzyme regulation. The structural insights gained from this study will pave way for large-scale screening of GDH regulators and could support researchers in the design and development of new and potent GDH ligands.

## 1. Introduction

Glutamate dehydrogenase (GDH) is a mitochondrial enzyme present in all organisms and plays an important role in glutamate metabolism by catalyzing the reversible oxidative deamination of glutamate to α-ketoglutarate (αKG) and ammonia [1,2,3,4,5,6,7]. Fungal GDHs exist as homotetramers, bacterial and vertebrate GDHs are homohexamers, and plant GDHs exist as homo- or heterohexamers [8,9]. Mammalian GDH is a homohexameric enzyme comprising two trimers stacked on top of each other. Each monomeric subunit consists of three major domains: (i) a substrate binding domain (Domain I), (ii) a coenzyme binding domain (Domain II), and (iii) the antenna domain (Domain III), which is absent in other GDH types, such as bacteria, fungi, plants, and most protists [9,10,11,12,13,14,15,16]. The antenna domain (helix–loop–helix) provides regulation by a wide range of ligands, including ADP (+), leucine (+), NADH (−), GTP (−), ATP (−), diethylstilbesterol (−), palmitoyl CoA (−), and steroid hormones (−), thus showing its importance in GDH modulation [9,17]. GDH is involved in a number of biological processes, including amino acid and carbohydrate metabolism, ammonia management, energy production, insulin secretion, and neurotransmitter recycling [18]. As a consequence, this enzyme is involved in a number of pathologies, such as cancer, central nervous system diseases, and hyperinsulinism/hyperammonemia syndrome; thus tight control of GDH is vital [19]. Previous studies have shown that this enzyme is modulated by a wide array of metabolites, including amino acids [20], antipsychotic drugs [21], nucleotides [22], natural products [23], and steroid hormones [24].

Although many studies related to the GDH enzyme have been conducted in the past, its modulatory mechanism remains elusive [3,5,10,11,25]. Several GDH structures have been solved, either crystallized with coenzymes (NADH, NADPH) and nucleotides (ADP, ATP, GTP and NADH) or mostly in its inactive form (closed state) [10,17,26,27]. Notably, crystalline or cryo-EM solved structures represent only a single snapshot, which are captured at a specific state. Moreover, the intrinsic dynamics of GDH states involving numerous allosteric regulators are yet to be elucidated. Additionally, intrinsic structural information in its active (open conformation) and apo states is still lacking. Therefore, it is necessary to study the dynamic nature of GDH complexes through molecular dynamic (MD) simulations, which could capture the protein motions in a time-dependent manner at an atomic scale resolution.

The conformational shift between ‘closed’ and ‘open’ GDH states is regulated by two allosteric sites which are differentially bound by inhibitor GTP and activator ADP, respectively. These allosteric regulators heavily regulate GDH function. ADP and GTP may bind to opposite conformational states. GTP binds to the hinge region when the Domain II (NAD^+^-binding domain) is in the closed conformation, and prevents the product release from the catalytic site through stabilization of the closed catalytic cleft, while the ADP-active site arises when the orthosteric site opens as Domain II pulls back towards the antenna region [28]. ADP acts as an activator by accelerating the catalytic cleft opening, resulting in the release of the reaction product [2,3,4,7,11,26,29,30,31].

Until now, only two computational studies on the GDH system have been published [19,32]. Recently, Rashid et al. utilized the available cryo-EM data of apo GDH, the open and closed states of NADH-, and GTP-bound GDH states to elucidate the allostery behind GDH regulation [19]. The results showed that the inhibitor triggers a triangular allosteric network linking GTP, NADH, and catalytic sites, thus regulating nucleotide-binding domain (NBD) motion, which blocks the catalytic cleft in the GTP-triggered inhibition dynamics. The authors combined cryo-EM data with MD (10 ns) and showed that the cofactor NADH is indeed a potent player in GDH modulation. However, signal communications, the key residues involved in ligand binding, allosteric communications, and conformational changes taking place in three different states of GDH have not been studied so far.

Here, we explored the conformational stability and mapped the signaling communications among the key residues for three different GDH states (i.e., the apo form of GDH without bound substrate; and the nucleotide-bound GDH states: the ADP-bound GDH state (agonist-bound GDH); and the GTP-bound GDH state without substrate and coenzymes (antagonist-bound GDH)) using MD simulations and network analysis. We carried out all-atom MD simulations for each GDH state (300 ns) and monitored their conformational structural and dynamical changes. Initially, the conformational stability of GDH complexes were assessed using MD structural analysis. Subsequently, we calculated the map of allosteric signal flow within GDH states by using “betweenness centrality” (*C_B_*), which is one of the most common analyses in network theory. Here, we assessed the betweenness centrality of each residue, calculated the maps of signal flow in GDH signaling, and identified hot spot residues vital for their modulatory mechanism. Interestingly, allosteric hot spots that were identified through *C_B_*-based analysis correlated well with previous biochemical and clinical mutation data [10,11,26,29,33,34,35]. Additionally, we also conducted a correlation network analysis for elucidating GDH enzyme-correlated motions in three different states. The structural underpinnings obtained from this study will pave way for large-scale screening of GDH modulators and could help researchers in the design and development of new and potent GDH ligands.

## 2. Materials and Methods

### 2.1. Molecular Dynamic Simulations of GDH States

The bovine GDH sequence shares 98% sequence identity to human GDH and 100% similarity in the near substrate and regulatory sites [29]. Bovine GDH serves as an excellent substitute for human GDH; hence, we utilized its crystallographic structures. By utilizing the X-ray crystal structures of bovine GDH states (apo form: 1NR7; agonist (ADP)-bound form: 1NQT; antagonist (GTP)-bound form: 3JD0), MD simulations were performed using the GROMACS package v5.1.4 [36] with a CHARMM36-jul2020 force field [37]. Since GDH is a homohexameric enzyme, we subjected only 1 monomer per GDH state to the MD simulations. Topology and parameter files for the ligands were generated via the CGenFF server v1.0.0 [38]. GDH states were solvated with 43,488 (apo), 29,917 (agonist-bound), and 33,303 (antagonist-bound) water molecules using the TIP3P water model. Subsequently, the states were neutralized by replacement of the solvent molecules with the required counter ions. The simulation states contained 138,183 (apo), 97,510 (agonist-bound), and 107,689 (antagonist-bound) atoms, respectively. Nonbonded interactions were smoothly switched off between 10 and 12 Å. Electrostatic interactions were handled using a particle mesh Ewald algorithm with a cubic interpolation order of 4 and a grid spacing of 0.16 nm.

Initially, GDH states were energy minimized using the steepest descent algorithm, where the tolerance force for convergence was set to <1000 kJ (mol nm)^−1^. Following energy minimization, the states were subjected to 2 equilibration phases (NVT (constant Number of particles, Volume, and Temperature) and NPT (constant Number of particles, Pressure, and Temperature)): (i) gradual heating of the system from 0 to 300 K with a 0.01 K interval at each step for 10 ns using a leap-frog integrator; (ii) subsequently, the systems were subjected to a second equilibration phase with the NVT ensemble for 10 ns using a Parrinello–Rahman barostat to reach 1 bar pressure. Upon completion of the 2-step equilibration, each system was subjected to a 300 ns production run using the NPT ensemble. We utilized an integration timestep of 2 fs, and the simulated trajectories were saved and sampled for every 100 ps for the analysis.

### 2.2. Structural Analysis

The trajectory produced for each GDH system after MD simulation was further analyzed using the built-in modules of GROMACS 5.1.4. [36,39] and plotted using matplotlib v3.3.2. The graphical images were produced using PyMOL v2.3.0a0 [40]. The structural analysis for each system was done using the gmx rms, gmx rmsf, gmx sasa, and gmx hbond utilities of GROMACS package to compute RMSD, RMSF, solvent-accessible surface area (SASA), and the number of hydrogen bonds (H-bonds). The distance criterion for the H-bonds is d ≤ 3.5 Å between the donor and acceptor, and the angle between the donor and acceptor is >30°.

### 2.3. Essential Dynamics

Principal component analysis (PCA) of MD simulations is a popular mathematical tool that computes the essential dynamics of a system on a low-dimensional free energy landscape (FEL) [41,42]. Covariance matrices for GDH states were calculated using protein backbone atomic fluctuations following the elimination of rotational and translational motions. From diagonalization of the covariance matrix, the sum of eigenvalues for apo, agonist-, and antagonist-bound GDH states were 66.584 nm^2^, 75.192 nm^2^, and 124.749 nm^2^, correspondingly. GROMACS tools, such as gmx covar and gmx anaeig, were used for retrieving the eigenvalues and eigenvectors of protein backbone atoms. The first 20 projection eigenvectors were subjected to cosine content analysis, where the principal components (PCs) with a cosine value of ≤0.2 were utilized for FEL calculations. The gmx sham module was used for retrieving the minimal energy configurations of each GDH state. Two-dimensional (2D) and 3-dimensional (3D) FEL contour plots were generated using a trial version of Wolfram Mathematica 12.2 [43].

### 2.4. Construction of the Residue Interaction Network

A weighted residue interaction network was constructed by considering each amino acid residue as a node and the number of H-bonds between two amino acid residues as a weight. To consider the effect of side chains, we included two coarse-grained centers per residue (the Cα backbone and the heavy atom furthest away from Cα for the side chain), so that the cases of backbone–side chain, side chain–side chain, and backbone–backbone contacts were encompassed. In our network model, if the distance between any 2 of the above-defined atoms in 2 residues was <7 Å, then a contact has been established.

### 2.5. Calculation of Network Centralities

In this study, we computed three centralities (*C_D_*, *C_C_*, and *C_B_*) from the residue interaction network. A brief description of each centrality is as follows: *C_D_* measures the number of contacts of each residue with the neighboring residues.

(1) $$C_{D}\left(r)=deg\;(r\right)$$

where *r* is the node.

*C_c_* calculates how fast a signal is transmitted from node *r* to other nodes.

(2) $$C_{C}(r)={\left(\overset{K}{\underset{j=1}{\sum }}d(q,\;r)/(K-1)\right)}^{-1}$$

where *K* is the chain length and d(q, r) is the number of edges that join the nodes *q* and *r*. We used Dijkstra’s algorithm to compute d(q, r).

*C_B_* measures the important residues involved in the signal transduction. We used Brandes algorithm to compute the *C_B_*.

(3) $$C_{B}=\frac{2}{\left(K-2)(K-1\right)}\overset{K-1}{\underset{a=1}{\sum }}\sum_{b=a+1}^{K}\frac{\tau_{ab}\left(r\right)}{\tau_{ab}}$$

where $\tau_{ab}$ is the shortest paths joining the nodes *a* and *b*, and $\tau_{ab}$ is the number of shortest paths connecting the nodes *a* and *b* through the node *r*.

### 2.6. Correlation Network Analysis

Correlation network analysis is used for detecting protein regions with correlated motions. In this method, a weighted graph is constructed, where each residue denotes a node and the weight of the connection between nodes *i* and *j* denotes their respective cross-correlation value, *cij*, as stated by the Pearson-like form [44] or the linear mutual information [45,46]. A detailed methodological description of computing a correlation network analysis from MD trajectories has been provided in previous studies [47,48]. The same procedure was implemented in Bio3D [49], which we utilized for computing the correlation networks.

## 3. Results

### 3.1. Structural Stability of GDH States

**Figure 1:**
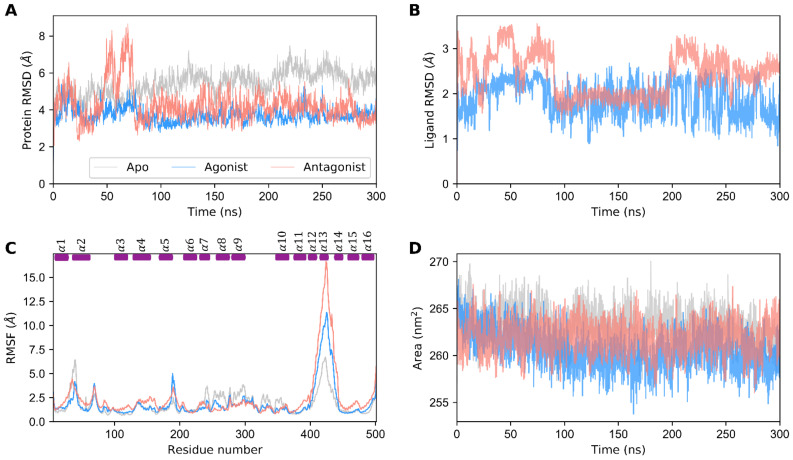
Structural stability analysis of apo, agonist-bound, and antagonist-bound GDH states. (**A**) The RMSD of the protein backbone atoms with respect to the equilibrated structure, (**B**) the RMSD of the agonist (ADP) and antagonist (GTP) molecules with respect to the minimized structures, and (**C**) the RMSF of the apo, agonist-, and antagonist-bound states of GDH are shown. Short rectangular boxes on the top axis indicate the location of the α-helices’ secondary structural elements. (**D**) Solvent-accessible surface area of the apo, agonist-, and antagonist-bound GDH states. The data for the apo, agonist-, and antagonist-bound forms of GDH are shown in gray, dodger blue, and salmon, respectively.

Apo, agonist-, and antagonist-bound GDH forms were prepared and subjected to 960 ns of MD simulations in total in an explicit aqueous solution. Initially, the stability of GDH states were evaluated using root mean square deviation (RMSD) of the protein backbone atoms with respect to the equilibrated structure in a time-dependent manner. Altogether, the three GDH states deviated from the equilibrated structure by 1.5 to 8 Å during a production run. As shown in Figure 1A, the apo state depicted high structural deviations varying from 1.5 to 7 Å, thereby depicting the structural instability of unbound states in the physiological state. Even though ligand-bound GDH states showed large structural deviations initially, both states reached stability after 75 ns. The agonist-bound GDH state (~5 Å) showed lower structural deviations than the antagonist-bound form. This clearly shows that the protein is more flexible in the antagonist-bound state than the agonist-bound form. Likewise, ligand RMSD was calculated for ADP (agonist) and GTP (antagonist) molecules (Figure 1B). RMSD for ADP ranged from 0.5 to 2.5 Å, while that for GTP ranged from 0.5 to 3.5 Å. In summary, not only the protein (agonist-bound state) but also the agonist (ADP) showed smaller RMSD when compared with the antagonist GTP.

Similarly, root mean square fluctuations (RMSFs) were calculated for the Cα atoms of GDH states (Figure 1C). Interestingly, the α2 and antenna helices (α12, α13, and α14) show high residual fluctuations. In particular, the antenna helix protein segment showed the maximal residual fluctuations in all three GDH states, thus underscoring its large intrinsic domain movement due to the unusual long protruding geometry, which plays a key role in enzyme regulation [11,19,29]. The fluctuations of the antenna helix for the antagonist-bound state (~16.5 Å) were much higher when compared with the agonist- and apo forms. Furthermore, the helices participating in ligand binding were comparatively stable and sustained their residual variations within 1 Å. Highly fluctuating regions for ligand-bound states were similar; however, the residual RMSFs of the agonist-bound state were much smaller than those of the antagonist-bound state. In one specific region (i.e., the loop connecting α5 and α6), the residue fluctutations of the agonist form were higher than those of antagonist and apo forms, which correlates well with our correlation network analysis (see Section 3.4.). Besides the α2 and antenna helices, the apo form showed residual fluctuations in the α8 and α9 helices. In addition to the RMSD, SASA was used to evaluate the stability of GDH states. Figure 1D shows that the agonist-bound state’s SASA is lower than that of the antagonist- and apo states, thus depicting its thermodynamic stability. The apo form showed higher SASA when compared with the agonist and antagonist-bound states. The average SASA for the apo, active, and inactive GDH states was 263.221 nm^2^, 260.551 nm^2^, and 261.953 nm^2^, respectively. Interestingly, the SASA values among the three states showed only marginal differences. Overall, our SASA analysis showed that all the three states were highly stable and did not vary over the course of the simulation.

**Figure 2:**
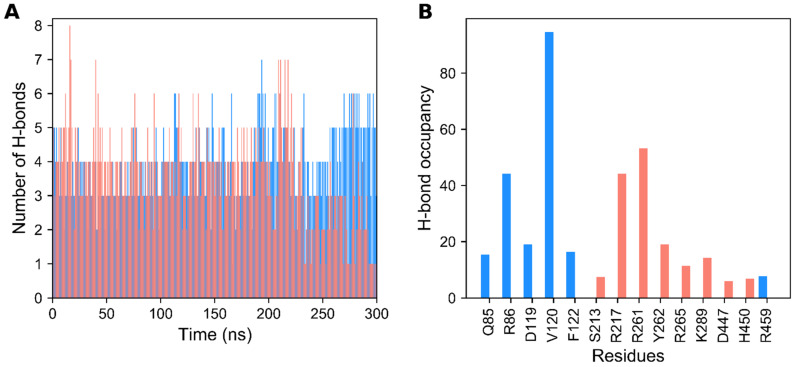
Hydrogen bond (H-bond) formation of agonist- and antagonist-bound GDH states during MD simulation. (**A**) Number of H-bonds formed between protein–ligand complexes during 300 ns of production time. (**B**) H-bond occupancy of each interacting residue in their relevant complexes throughout the simulation. The data for the agonist- and antagonist-bound forms of GDH are shown in dodger blue and salmon, respectively.

Besides the RMSD and SASA analyses, we also checked the stability of hydrogen-bonds (H-bonds) in the agonist- and antagonist-bound GDH states throughout the simulation. In Figure 2A, it can be observed that the number of H-bonds in the ligand-bound states varied throughout the course of the MD simulation. The average number of H-bonds per timeframe for the ligand-bound states were 3.482 and 4.118, respectively, for the agonist- and antagonist-bound states. The maximum number of H-bonds in ligand-bound states were, respectively, 7 and 8 for the agonist- and antagonist-bound states. Approximately three H-bonds were retained in the ligand-bound states throughout the simulations. Moreover, critical residues that were involved in H-bond formation for ligand recognition were identified using H-bond occupancy. H-bond occupancy can be defined as the fraction of time involved in H-bond formation between the ligand and protein atoms. In Figure 2B, it is shown that the Q85, R86, D119, V120, and F122 residues mainly contribute to H-bond formation in the agonist-bound state, which is in line with the previous reports [10]. Additionally, the R459 residue contributes minorly to H-bond formation, which plays an important role in ADP activation, as the mutant form of R459A leads to a loss in ADP activation [10,11]. However, in the antagonist-bound state, R217, R261, Y262, R265, and K289 are the major residues involved in H-bonding formation, which agrees with previous studies [10,11]. Other minor residues include S213, D447, and H450. In brief, H-bond analysis shows the prominence of polar interactions in the selective recognition of GDH ligands.

### 3.2. PCA and FEL Analysis

**Figure 3:**
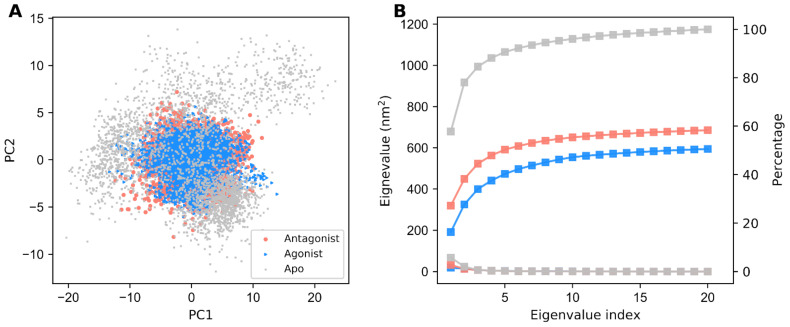
Principal component (PC)1 and PC2 projections of the different structural conformations of GDH states. (**A**) Two-dimensional (2D) projection of PC1 and PC2 throughout the MD simulations for the apo, agonist-bound, and antagonist-bound GDH states, which are represented in gray, dodger blue, and salmon, respectively. (**B**) The best PCs of eigenvalues corresponding to the first 20 eigenvectors and their related cumulative fluctuations are represented by squares.

PCA was used for reducing the dimensionality of the data while preserving most of the variation in the data [50]. Here, we utilized essential dynamics analysis (EDA) to study the distribution of the structural conformations among three different GDH states from the MD simulation trajectories. Two-dimensional projections of PC1 and PC2 between the largest two eigenvalues in the GDH states are mapped in Figure 3A. The conformation of each individual GDH state over a trajectory of 300 ns is represented by dots. Notably, if a protein showed large conformational changes, then the distribution was also scattered in the conformational space. In our EDA analysis, the apo form’s distribution is more widely dispersed than ligand-bound forms due to the absence of a ligand. However, the distribution of GDH ligand-bound states in the conformational space is limited.

Additionally, we plotted eigenvalues against the eigenvector index and identified that all GDH states displayed stabilization in the first eight eigenvectors, as depicted in Figure 3B. We also computed the cumulative percentages of variance in motion retrieved from the initial 20 PCs (91%, 51%, and 56% for the apo, active, and inactive forms, respectively). Overall, the PCA results suggested that the apo form of GDH showed a large structural distribution when compared with the active and inactive forms. The active form showed less structural distribution when compared with the inactive form, thereby showing the highly stable nature of GDH upon agonist binding.

**Figure 4:**
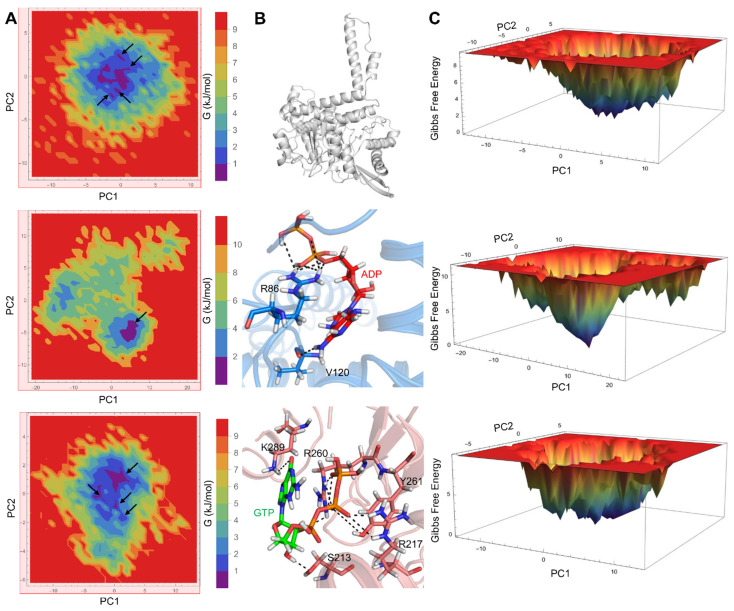
Free energy landscape (FEL) contour plots of GDH states. (**A**) Two-dimensional FELs of apo (top panel), agonist-bound (middle panel), and antagonist-bound (bottom panel) GDH states, shown as a function of two PCs that were selected based on a cosine content value ≤0.2. The minimal energy clusters in all three GDH states are shown by black arrows. (**B**) The minimal energy structures of apo, ADP-bound, and GTP-bound GDH states are shown. Residues critical for ligand recognition are depicted as blue and salmon-colored sticks and H-bonds are shown as black dashed lines. (**C**) Three-dimensional FEL plots of apo (top panel), agonist-bound (middle panel), and antagonist-bound (bottom panel) GDH states.

We also conducted FEL analysis to obtain the global minimum energy conformation of GDH states. Two-dimensional and 3D FEL contour maps for the GDH states were obtained from PC1 and PC2 (Figure 4). The 2D-FEL contour maps for ligand-bound GDH states presented four minimal energy clusters and larger structural dispersals (Figure 4A). A single cluster was observed in the agonist-bound GDH state, thus demonstrating the robust and stable interaction of ADP with GDH, which is in line with our structural stability analysis. In Figure 4B, it can be observed that we extracted the most representative conformation from the least minimal energy regions in the agonist- and antagonist-bound GDH states to check the ligand interactions in the active site. FEL analysis of the ligand-bound states (Figure 4C) showed that the lowest energy conformers were identified at the 198,200 ps (agonist-bound state) and 150,200 ps (antagonist-bound state) snapshots retrieved from the most populated free energy minimum cluster. Key ligand interactions in the agonist- and antagonist-bound GDH states, as shown in Figure 4B, correlated well with previous reports [10,11,17,18,29].

### 3.3. Identification of GDH Hot Spot Residues Using Network Analysis

**Figure 5:**
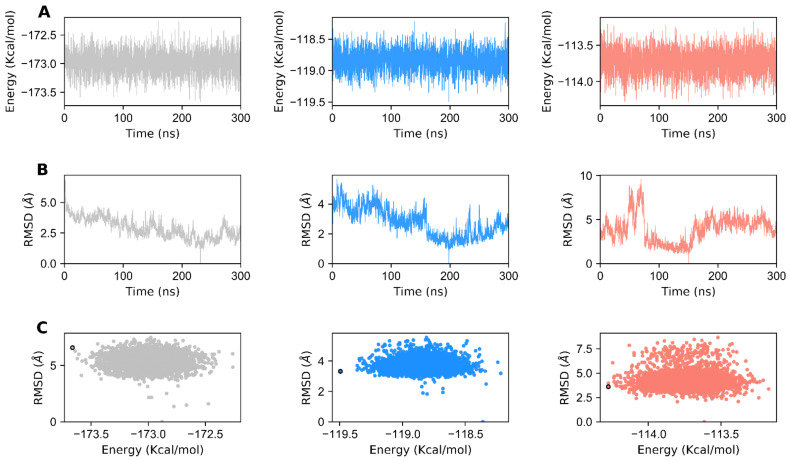
Generation of minimal energy structures of GDH for network analysis. (**A**) Time-dependent total conformational energy of apo, agonist-, and antagonist-bound GDH states. (**B**) RMSD with reference to the minimal energy structure from the MD trajectories of GDH states. (**C**) Total conformational energy versus RMSD plots for apo, agonist- and antagonist-bound GDH complexes. The minimal energy structures of GDH states are shown as black circles. The data for apo, agonist-and antagonist-bound forms of GDH are shown in gray, dodger blue, and salmon, respectively.

Choosing a representative structure from a long MD trajectory for analysis is a daunting task. However, choosing the last frame as the representative structure does not ensure that it will be the best structure with the lowest energy score. One better alternative would be to cluster the trajectories and subsequently consider the centroids of the most populated cluster as the representative one. Nevertheless, the choice of a representative selection criterion would depend on the goal of our analysis. Here, we monitored each state in terms of total conformational energy (Figure 5A) and RMSD relative to the minimal energy structure (Figure 5B). The representative structures for each GDH state were obtained by plotting the total conformational energy relative to the RMSD of the backbone atoms of the minimal energy structure (Figure 5C). The top 20 structures obtained from the conformational ensemble with the lowest energies were clustered for the apo, agonist-, and antagonist-bound GDH forms. The most minimal energy structure for each state was chosen as the representative one, and was subjected to network analysis. GDH monomers’ structural architecture and the structural differences observed in the most minimal energy structures of the apo, agonist-, and antagonist-bound states are shown in Figure S1.

**Figure 6:**
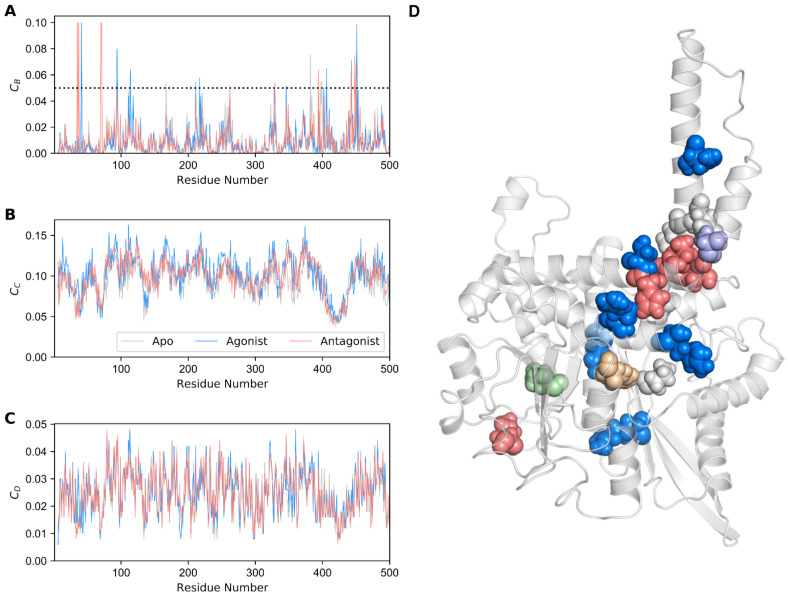
Network centrality analysis for GDH states. (**A**) Betweenness (*C_B_*), (**B**) closeness (*C_c_*), and (**C**) degree (*C_D_*) centralities for each residue of the GDH complexes are shown. The dotted line represents the cutoff value *C_B_* = 0.05. (**D**) Residues with high *C_B_* (≥ 0.05) are depicted as spheres on the minimal energy structure of apo GDH. Gray, dodger blue, and salmon spheres represent the residues with high *C_B_* values in the apo, agonist-, and antagonist-bound GDH states, respectively. Other residues that are commonly present in the apo and agonist-bound GDH states, the apo and antagonist-bound GDH states, and the agonist- and antagonist-bound GDH states are shown in pale green, wheat, and purple, respectively.

Network centrality is used as a popular method for elucidating protein allostery. In network theory, this statistical measure quantifies the degree of centralization of a node, which could be utilized to elucidate the protein hot spot residues [51]. We computed centrality measures, such as betweenness (C_B_), closeness (C_C_), and degree (C_D_) centralities [52], by using the minimal energy structures of all three GDH forms (Figure 6A–C), built residue interaction network as stated in Materials and Methods section, and calculated the GDH states’ centralities. We considered only betweenness centrality to assess the significance of each residue for the signal transduction in the GDH forms. The rationale for using the C_B_ measure has been discussed in our previous work [42].

Residues with C_B_ ≥ 0.05 in all three GDH states were mapped onto the minimal energy apo structure (Figure 6D). A total of 20 hot spot residues were identified, of which six were detected in the apo state, eight in the agonist-bound state, and six in the antagonist-bound GDH state (Table 1). Among them, a few residues were deemed to be important in signaling, which is in line with previous studies [10,11].

In the apo form, residues with C_B_ ≥ 0.05 were found in the α8, α11, and α12 helices. Similarly, residues with C_B_ ≥ 0.05 in the antagonist-bound state were identified in the α8, α12, and α15 helices. In the agonist-bound state, the residues with C_B_ ≥ 0.05 were found to be distributed in the α3, α6, α12, α14, and α15 helices. Interestingly, most of the residues were identified to be well distributed in the antenna region in all three states. However, in the ligand-bound states, the residues were well dispersed in the pivot helix (α15) as well. Overall, the residues identified through this analysis were well distributed in the α3, α6 (intermediate), α8, α11 (elongated), α12, α14 (antenna), and α15 (pivot) helices, and thus could be deemed important for modulating GDH signaling.

**Figure 7:**
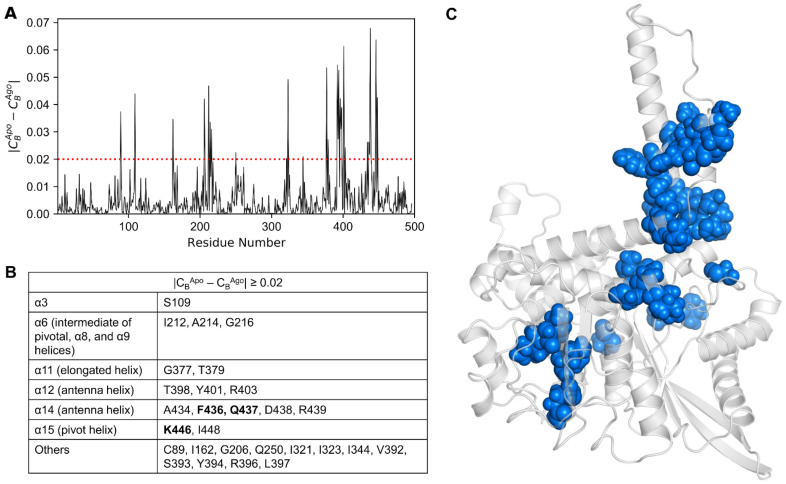
(**A**) Differences in *C_B_* values calculated for the apo and agonist-bound GDH states. (**B**) Key residues satisfying the condition |*C_B_^Apo^* − *C_B_^Ago^*| ≥ 0.02, along with their corresponding protein segments (listed in the table). Mutation data available for residues are shown in bold. (**C**) Residues with |*C_B_^Apo^* − *C_B_^Ago^*| ≥ 0.02 are shown on the minimized apo structure as dodger blue spheres.

Additionally, we computed the differences in C_B_ between the apo and agonist-bound (Figure 7A), and the apo and antagonist-bound states. In total, 28 residues that satisfied |C_B_^Apo^ − C_B_^Ago^| ≥ 0.02 were identified (Figure 7B) and mapped onto the minimal energy structure of the apo form (Figure 7C). In Figure 7B,C, it is clear that the residues satisfying the condition |C_B_^Apo^ − C_B_^Ago^| ≥ 0.02 are primarily distributed in the α3, α6, α11, α12, α14, and α15 alpha helices. Besides the helices, a few of the residues are well dispersed in the loop regions, mostly in the α11–α12 loop.

**Figure 8:**
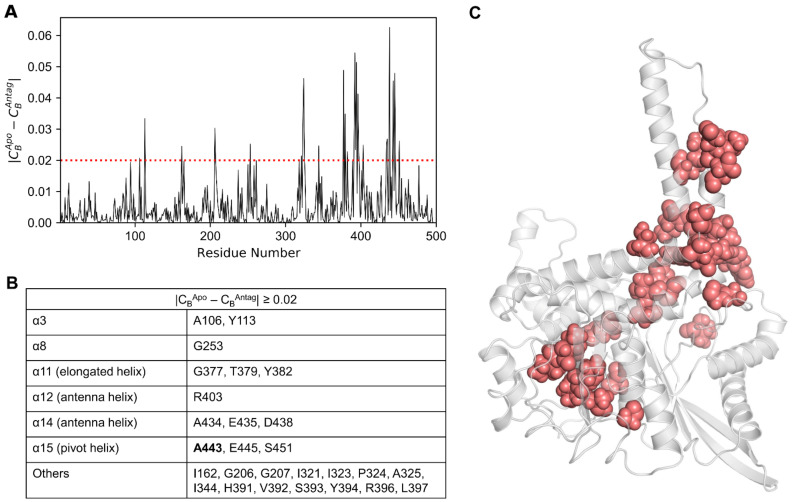
(**A**) Differences in *C_B_* values calculated for the apo and antagonist-bound GDH states. (**B**) Key residues satisfying the condition |*C_B_^Apo^* − *C_B_^Antag^*| ≥ 0.02, along with their corresponding protein segments (listed in the table). Mutation data available for residues are shown in bold. (**C**) Residues with |*C_B_^Apo^* − *C_B_^Antag^*| ≥ 0.02 are shown on the minimized apo structure as salmon spheres.

In the case of the |C_B_^Apo^ − C_B_^Antag^| ≥ 0.02 condition (Figure 8A), a total of 27 residues were identified (Figure 8B), which were mapped onto the lowest energy apo structure (Figure 8C). Here, the residues were located in the α3, α8, α11, α12, α14, and α15 helices. Few residues were distributed along the loop regions, mostly in the α9–α10 and α11–α12 loops. The contribution of residues satisfying both conditions were mainly identified in the α3, α6, α8, α11, α12, α14, and α15 pivot helices, which agreed well with the C_B_ ≥ 0.05 condition. The predictions derived from C_B_-based network analysis are useful not only for complementing mutagenesis studies but also for illuminating the modulatory mechanisms of the GDH enzyme.

### 3.4. Correlation Network Analysis of GDH States

**Figure 9:**
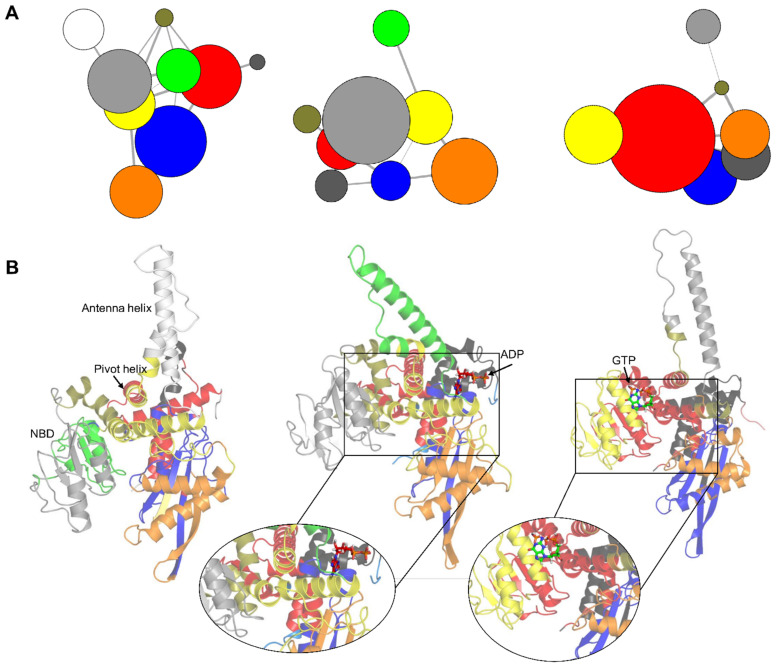
Correlation network analysis of the apo, agonist-, and antagonist-bound GDH states. (**A**) Community networks of the apo, agonist-, and antagonist-bound GDH states are shown as colored circles whose radius depicts the number of residues in a specific community. The linking lines represent the intercommunity couplings. Five major correlated protein regions (red, light gray, orange, blue, and yellow) were identified in all three GDH states. (**B**) Protein regions of the apo, agonist-and antagonist-bound states corresponding to each community are shown, and the colors match the community partitioning. In the apo form, the five main correlated protein sectors are blue (α2, α2–α3 loop, α4–α5 loop, α5, and α9–α10 loop), red (α1, α2, α2–α3 loop, α15 (pivot helix), and α16), light gray (α7 → α9, and α9–α10 loop), orange (α2–α3 loop, α4, α5, and α5–α6 loop), and yellow (α5–α6 loop, α10–α11 loop, α11, α14, and α15). In the agonist-bound GDH state, the five main correlated community groups are light gray (α6 → α9, and α9–α10 loop), orange (α2–α3 loop, α4, α5, and α5–α6 loop), yellow (α4–α5 loop, α5–α6 loop, α6, α11, and α14), red (α1, α3, α6, α10–α11 loop, α15, and α16), and blue (α2, α2–α3 loop, α3, and α3-α4 loop). The five major correlated protein sectors in the antagonist-bound GDH state are red (α1, α3, α5–α6 loop, α6, α7–α8 loop, α8, α10, α11, α15, and α16), yellow (α5–α6 loop, α10–α11 loop, α11, α14, and α15), blue (α2, α2–α3 loop, α3, α3–α4 loop, α4, and α4–α5 loop), orange (α2–α3 loop, α3–α4 loop, α4, α4–α5 loop, α5, α6, and α11), and dark gray (α2, α2–α3 loop, α3, and α16). The zoomed-in views show the participating major communities in the ligand binding regions.

To interpret and quantify GDH state-specific couplings, correlation networks were constructed. This method has been successfully implemented to elucidate allosteric couplings in several states [48,53,54,55]. For each GDH state, a weighted correlation network graph was constructed from the MD simulations. Each node represented individual protein residues and the weight of the connection between nodes represented their related cross-correlation value. We computed correlation networks using MD simulation trajectories and applied community analysis to cluster the networks into highly intra-correlated structural regions, but loosely connected substructures. This analysis revealed nine, eight, and seven correlated protein regions (or community groups) in the apo, agonist-, and antagonist-bound GDH states, respectively (Figure 9A).

In the apo form, there were five main correlated protein sectors (Figure 9B), shown as blue, red, light gray, orange, and yellow. In the agonist-bound GDH state, five main correlated community groups (Figure 9B), shown as light gray, orange, yellow, red, and blue, were detected. Similarly, five major correlated protein sectors were identified in the antagonist bound GDH state (Figure 9B), shown as red, yellow, blue, orange, and dark gray. The results suggest that the participating protein segments showed an intrinsic tendency to demonstrate correlated motions regardless of the GDH state. In the ADP-bound form, active site residues were distributed in major communities (blue, red, and yellow) (Figure 9B). Likewise, antagonist-bound GDH inhibitor binding site’s residues were dispersed in the major red and yellow communities (Figure 9B).

## 4. Discussion

GDH is an ubiquitous enzyme that plays an important role at the branchpoint of the nitrogen and carbon assimilation pathways [9]. Animal GDH is regulated by a wide range of allosteric ligands, including ADP and GTP, which interact in an agonistic and antagonistic manner, respectively. Since GDH is modulated by several metabolites, the allosteric communication taking place within the GDH intra- or interdomain must be elucidated. Several studies related to GDH regulation have been investigated in the past; however, the underpinnings of GDH’s modulatory mechanism at an atomic level remain elusive. Even though several GDH structures have been solved in the closed state, apo or open forms have been less investigated. Hence, we applied unbiased MD simulations to apo, active ADP-bound, and inactive GTP-bound GDH forms for elucidating the ligand recognition mode and their modulatory mechanisms. We subjected three GDH states to MD simulations for a total of 960 ns and inspected their trajectories utilizing structural stability and network analyses.

Initially, we assessed the structural stability of GDH states using RMSD, RMSF, SASA, and H-bond analysis. Our structural stability analysis identified the more stable nature of the agonist-bound GDH state in comparison with the apo and inactive forms. RMSF analysis identified high residue fluctuations in the α2 and antenna helices, thus indicating the importance of these regions in GDH’s modulatory process. In particular, the antagonist-bound GDH state showed high residue fluctuations in the antenna helix region in comparison with the other two GDH states, thus showing its significance in the regulation process in a closed state. SASA analysis also supported the stable nature of the three GDH states. Furthermore, H-bond analysis illustrated the importance of polar interactions and identified the major and minor residues involved in ligand recognition, which agrees well with previous studies [11,26,43,53,54]. EDA analysis also supported the stable nature of ligand-bound GDH states in comparison with the apo form, which is in line with H-bond analysis and previous studies [6,11,45,46,55]. PCA showed the widely scattered structural distribution of the apo form due to the absence of ligands. Overall, our structural and EDA analyses showed the importance of specific regions in ligand recognition and the modulatory mechanism, and strongly supported the stable nature of GDH states.

Furthermore, we applied the *C_B_* measure to all GDH states for evaluating the significance of each residue in mediating the signal flow. The residues identified through network analysis correlated well with the experimental and mutation data [11,19,21,24,29,38]. We also constructed correlation network analysis to quantify GDH-state-specific couplings and identified that the major clusters were identified in the active site, NBD, intermediate, extended, and pivot helices which are deemed to be important for GDH signaling [11,29]. The predictions derived from the *C_B_*-based network and correlation network analysis provides a better insight of the intricate links among allostery, protein flexibility, and function. This study identified a few hot spot residues and important protein regions which could be utilized in future investigations for capturing their local dynamics in the GDH signaling process. For future prospects, this investigation could be extended by subjecting the monomer or oligomer to microsecond simulations and identifying the key microswitches which could play important roles in enzyme modulation. Our approach should be of great use not only for investigating ligand recognition studies of the GDH state but also for elucidating their signaling and modulatory mechanisms. This approach could be extended for studying and capturing both the local and global dynamics of protein–ligand states and to map their coupled signal flow.

## Figures and Tables

**Table 1:** List of important residues satisfying the condition (*C_B_*) ≥ 0.05 are shown. Mutation data available for residues are shown in bold. Residues with |*C_B_*^Apo^ − *C_B_*^Ago^| ≥ 0.02 are underlined. Residues with |*C_B_*^Apo^ − *C_B_*^Antag^| ≥ 0.02 are italicized.

***C_B_* ≥ 0.05**	
*C_B_*^Apo^ ≥ 0.05	P167, *Y262, ^#^E346, Y*382*, *L397*, F399
*C_B_*^Ago^ ≥ 0.05	R94, K114, R211, **R217**, ^#^E346, **N406**, ^&^***A443***, *S451*
*C_B_*^Antag^ ≥ 0.05	*Y262, K329, Y*394*, ^&^***A443***, I448, **S450**

* Residue with *C_B_* ≥ 0.05 present in both the apo and antagonist-bound forms. ^#^ Residue with *C_B_* ≥ 0.05 present in both the apo and agonist-bound forms. ^&^ Residue with *C_B_* ≥ 0.05 present in both the agonist- and antagonist-bound forms.

## Supplementary Materials

The following are available online at https://www.mdpi.com/article/10.3390/biom11060798/s1. Figure S1. Structural architecture and superposition of the GDH monomer structure; M1 Supporting Movie: Conformational changes observed in the apo GDH state during 300 ns of simulation time; M2 Supporting Movie: Conformational changes observed in the agonist-bound GDH state during 300 ns of simulation time; M3 Supporting Movie: Conformational changes observed in the antagonist-bound GDH state during 300 ns of simulation time.
