# Supplementary material for: Mapping the Intramolecular Communications among Different Glutamate Dehydrogenase States Using Molecular Dynamics
Source: Biomolecules. 2021 May 27;11(6):798. doi: 10.3390/biom11060798 (PMC8228935; doi:10.3390/biom11060798)
Supplement: Supplementary file 1 [file biomolecules-11-00798-s001.zip › biomolecules-1205428 suppl orig.pdf]

Supporting Information

Mapping the Intramolecular Communications Among Different Glutamate Dehydrogenase States using Molecular Dynamics

Shaherin Basith [1], Balachandran Manavalan[1], Tae Hwan Shin[1], and Gwang Lee [1,2,*]

[1]Department of Physiology, Ajou University School of Medicine, Suwon, Republic of Korea; sh aherinb@gmail.com, bala@ajou.ac.kr

[2]Department of Molecular Science and Technology, Ajou University, Suwon, Republic of Korea; glee@ajou.ac.kr

*Corresponding author: Gwang Lee

Professor, Department of Physiology

Ajou University School of Medicine, 164, World cup-ro,

Yeongtong-gu, Suwon, 16499, Republic of Korea.

Tel: +82-31-219-4554

Fax: +82-31-219-5049

E-mail: glee@ajou.ac.kr

Supplementary Figure 1

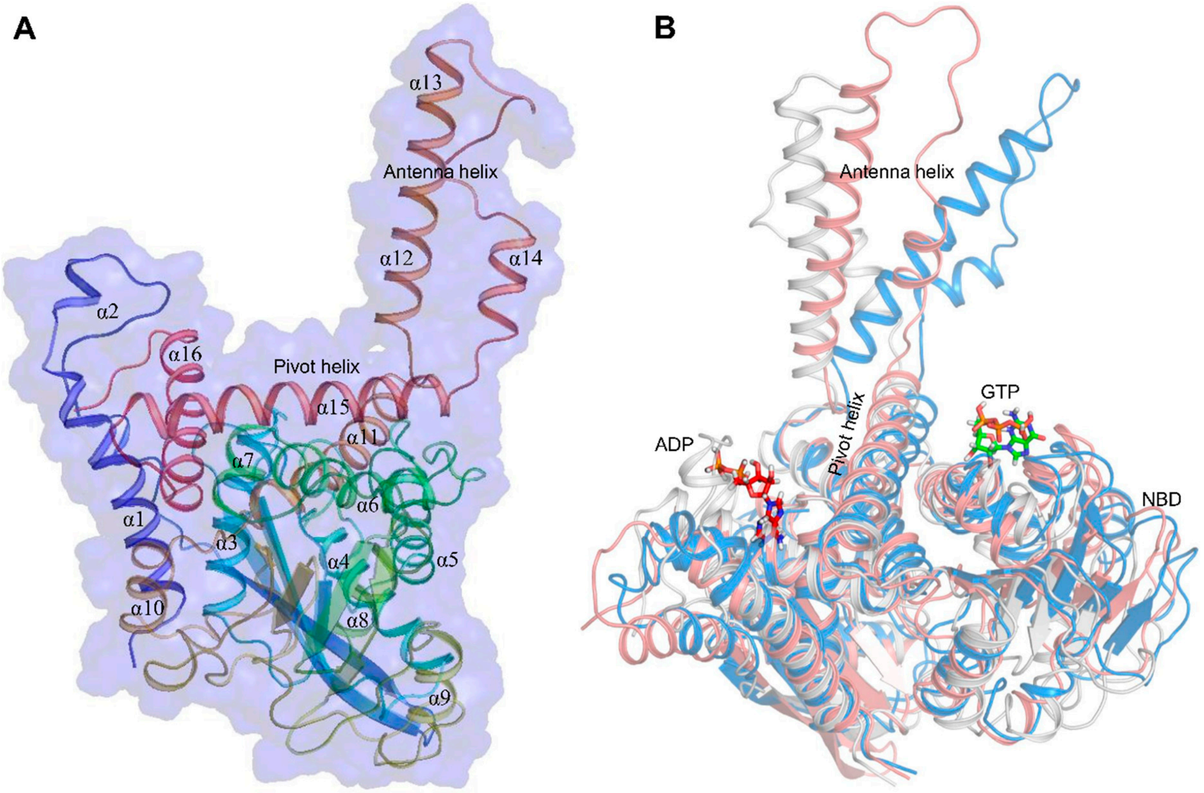

Figure S1. Structural architecture and superposition of GDH monomer structure. (A) Sixteen α-helices including intermediate, elongated, antenna, and pivot helices, and conserved nucleotide-binding domain (NBD) is shown. (B) The minimum energy structures of GDH in the apo, agonist- and antagonist-bound forms are superimposed to show that the most significant differences among the three forms is in the ligand binding regions, NBD, intermediate helix (α6), elongated helix (α11), antenna helices, and pivot helix.
